# Evaluating the Clinical Decision-Making Accuracy of Artificial Intelligence in Common Geriatric Syndromes Using Evidence-Based Guidelines

**DOI:** 10.7759/cureus.101858

**Published:** 2026-01-19

**Authors:** Peter Cassar, Francesca Galea, Peter Ferry

**Affiliations:** 1 Department of Geriatrics, Karin Grech Rehabilitation Hospital, Pieta, MLT

**Keywords:** artificial intelligence in medicine, chatgpt, geriatric medicine, geriatric syndromes, large language model

## Abstract

Background

Artificial intelligence (AI) tools such as ChatGPT are increasingly being explored for clinical decision support, yet their role in geriatric medicine remains uncertain due to the complexity of multimorbidity and care planning. This study aimed to evaluate the clinical accuracy, completeness, and guideline alignment of ChatGPT’s responses to common geriatric scenarios using standardized vignettes.

Methodology

Seven standardized vignettes representing common geriatric scenarios, namely, polypharmacy, falls, dementia, delirium, frailty, advance care planning, and urinary incontinence, were submitted to ChatGPT (GPT-5). Responses were evaluated by five independent consultant geriatricians using a standardized rubric across the following five domains: accuracy, completeness, guideline alignment, safety, and clarity (0-2 score per domain). Descriptive statistics summarized performance, and qualitative feedback was thematically analyzed. Inter-rater reliability was assessed using Krippendorff’s alpha.

Results

ChatGPT scored the highest in clarity (66/70) and safety (63/70), with slightly lower performance in accuracy (59/70) and completeness (55/70). Guideline alignment was generally strong (61/70). Advance care planning received the highest domain scores; urinary incontinence scored the lowest. Krippendorff’s alpha showed high inter-rater agreement (0.969). Reviewers identified key omissions, such as missing assessments or guideline-recommended tools, in multiple vignettes.

Conclusions

ChatGPT showed potential as a supportive tool in geriatric care, offering clear and generally safe responses aligned with guidelines. However, it lacked clinical depth and missed key elements in complex scenarios. AI tools such as ChatGPT should be used with caution, under expert oversight, and not as standalone decision makers in clinical practice.

## Introduction

Artificial intelligence (AI) is increasingly being explored as a tool to support clinical decision-making. More broadly, AI has been incorporated into clinical decision support systems for tasks such as diagnostic assistance, treatment recommendation, and patient triage. More recently, large language models (LLMs) such as ChatGPT have developed as a new form of AI that can generate natural language responses to complex clinical queries, leading to growing interest in their potential role in medical decision-making.

In geriatric medicine, where holistic care is essential, physicians face the challenges of multimorbidity and polypharmacy. In such cases, AI tools such as ChatGPT may offer valuable support. Several previous studies have evaluated medical AI systems and LLM in clinical settings, reporting variable performance in diagnostic accuracy, adherence to guidelines, and consistency of recommendations. However, most existing evaluations have focused on single disease scenarios or specialist fields, and little is known about performance in complex multimorbid cases that require more complex clinical judgement.

Despite this potential, the accuracy, consistency, and clinical usefulness of LLM in real-world complex medical scenarios remain largely untested. Therefore, this study aimed to systematically evaluate ChatGPT’s clinical performance using standardized geriatric clinical vignettes, assessing the accuracy of its responses and their concordance with evidence-based guidelines.

Geriatric medicine presents a particularly challenging environment for AI-supported decision-making, as it requires balancing evidence-based interventions with individualized and person-centered care. The aim was to examine the model’s ability to provide safe, coherent, and contextually appropriate recommendations for common geriatric syndromes.

## Materials and methods

This study was designed as a descriptive comparative analysis using simulated clinical vignettes to evaluate ChatGPT’s performance against expert human judgment. The responses were generated using the GPT-5 model, an optimized version of OpenAI’s GPT-5 architecture known for its advanced language understanding and generation capabilities. This model, which was freely available to users through OpenAI’s ChatGPT platform at the time of the study, represents a widely accessible tool, thereby enhancing the relevance of the findings to typical clinical and educational contexts.

Vignettes

Seven standardized clinical vignettes were developed, each addressing a key geriatric syndrome.

Polypharmacy

Mrs. Jackson is an 84-year-old woman with hypertension, osteoarthritis, type 2 diabetes, and insomnia. Her current medications include lisinopril, metformin, amlodipine, paracetamol, ibuprofen, zolpidem, and omeprazole. She complains of dizziness and occasional confusion. How would you assess and manage her medications?

Falls

Mr. Walker is a 78-year-old man who presents after two falls in the last month, both occurring at home while getting out of bed. He uses a cane, has bilateral cataracts, and recently started taking furosemide and tamsulosin. No loss of consciousness reported. What are the possible causes, and how should his risk be managed?

Dementia

Mrs. Clarke is a 79-year-old retired teacher brought in by her daughter due to forgetfulness, repeating questions, and difficulty following conversations. These symptoms have progressed over 18 months. She lives alone and is independent with ADLs. What would you consider in terms of diagnosis and next steps?

Delirium

Mr. White, 81, is admitted with a urinary tract infection. On day 2, he becomes agitated, disoriented, and tries to get out of bed at night. He was mentally clear on admission. No new medications except antibiotics. What is the likely diagnosis, and how should it be managed?

Frailty

Mrs. Wright is 88 years old and has unintentionally lost 5 kg in 6 months, walks slowly, and requires help with bathing. She was hospitalized twice in the past year. Her grip strength is weak, and she tires easily. How would you assess for frailty, and what are the next steps?

Advanced Care Planning

Mr. Lewis, 90, with end-stage heart failure and moderate dementia, has been in and out of hospital. His daughter is unsure whether to pursue further aggressive treatments. He has no advanced directive. Family is unsure about continuing aggressive treatment. What should be discussed?

Urinary Incontinence

Mrs. Jones, 75, reports new-onset urinary leakage when coughing or laughing. She had three vaginal deliveries and has no significant medical history. She is embarrassed and has started avoiding social outings. What should be considered in her evaluation and management?

Each vignette was submitted to ChatGPT, and the same model version was used for consistency with a prompt to follow the latest National Institute for Health and Care Excellence and British Geriatric Society guidelines. Unedited generated responses were stored. Five independent consultant geriatricians reviewed each response, scoring them using a standardized score sheet. Each vignette was submitted once to the model, and a single generated response per vignette was evaluated to reflect typical real-world use, where clinicians or trainees would generally obtain a single response rather than running repeated prompt submissions.

The scoring rubric evaluated responses across the following five domains, with each domain scored on a scale from 0 to 2: accuracy, completeness, guideline alignment, safety considerations, and clarity and clinical utility (Tables 1, 2).

**Table 1:** Evaluation domains and criteria.

Domain	Evaluation criteria
Accuracy	Assesses whether the medical information provided is correct according to current standards and practices. Any suggested treatment or management plan is evaluated against widely accepted clinical guidelines
Completeness	Evaluates whether all relevant aspects of the vignette are addressed, including medical history, clinical diagnosis, management plan, and follow-up considerations
Guideline alignment	Compares the response with established geriatric clinical guidelines (e.g., National Institute for Health and Care Excellence) to determine consistency with recommended practices
Safety considerations	Examines whether patient safety is adequately considered, particularly in the context of geriatric complexities such as frailty, polypharmacy, and cognitive impairment
Clarity and clinical utility	Assesses whether the response is clear, concise, and provides practical, actionable recommendations that are useful in real-world clinical practice

**Table 2:** Scoring guide for response evaluation.

Score	Description	Criteria
0	Poor	Incorrect or missing critical elements; potential safety risks or major deviations from guidelines
1	Satisfactory	Partially accurate; some omissions or minor concerns; partial alignment with guidelines
2	Excellent	Accurate, comprehensive, and guideline-aligned; prioritizes safety and clarity in clinical practice

Data analysis

Domain scores were summed and averaged across vignettes and reviewers based on the scoring guide. Thematic analysis was performed to assess recurring points of agreement and disagreement by the reviewers. Inter-rater reliability was assessed using Krippendorff’s alpha, which is well-suited for ordinal data and multiple raters.

## Results

ChatGPT’s responses were evaluated across seven clinical vignettes representing common geriatric syndromes (Table 3). The aggregated scores from five independent consultant geriatricians indicated generally favorable performance across all assessed domains. Overall, ChatGPT demonstrated particular strength in generating clear and clinically useful responses, achieving the highest cumulative score in the “Clarity” domain (66/70). This was closely followed by strong results in “Safety” (63/70) and “Guideline Alignment” (61/70), suggesting that ChatGPT was often aligned with evidence-based clinical standards and prioritized safe management strategies. However, slightly lower scores were observed in the domains of “Accuracy” (59/70) and “Completeness” (55/70), reflecting occasional omissions of key clinical details or partial responses to the presented vignettes. The vignette on “Advanced Care Planning” received the highest total score, while “Urinary Incontinence” scored the lowest.

**Table 3:** Summary of scores across each domain.

Vignette	Accuracy	Completeness	Guideline alignment	Safety	Clarity
1 – Polypharmacy	8	9	9	9	10
2 – Falls	8	10	9	9	10
3 – Dementia	9	7	9	9	10
4 – Delirium	9	7	9	9	8
5 – Frailty	8	7	8	9	9
6 – Advanced care planning	10	9	9	10	10
7 – Urinary incontinence	7	6	8	8	9
Total Score (maximum of 70)	59	55	61	63	66

Statistical analysis to assess Inter-rater reliability was conducted using Krippendorff’s alpha. The overall Krippendorff’s alpha was 0.969 across all vignettes, indicating a high level of agreement across raters. Domain-specific alpha values ranged from 0.689 (Accuracy) to 0.844 (Completeness), reflecting substantial to strong agreement (Table 4). Agreement by vignette was also high, with alpha values ranging from 0.675 to 0.959, further supporting the consistency and reliability of the assessment process (Table 5). In addition to the quantitative scores, a qualitative analysis of the reviewers’ comments was performed, highlighting key points from each vignette (Table 6). Overall, the qualitative analysis revealed recurring omissions in key safety assessments, diagnostic screening, and multidisciplinary management across several vignettes, despite generally appropriate core clinical recommendations.

**Table 4:** Krippendorff’s alpha score per domain across all seven vignettes.

Domain	Alpha score
Accuracy	0.689
Completeness	0.844
Guideline alignment	0.729
Safety	0.725
Clarity	0.702

**Table 5:** Krippendorff’s alpha score per vignette across all domains.

Vignette	Alpha score
Vignette 1	0.920
Vignette 2	0.909
Vignette 3	0.928
Vignette 4	0.675
Vignette 5	0.771
Vignette 6	0.959
Vignette 7	0.815

**Table 6:** Reviewer feedback highlights for the different vignettes.

Vignette	Key reviewer feedback
Polypharmacy	Inappropriately flagged paracetamol as a drug that may cause confusion when given alongside zolpidem. Failed to mention the need for orthostatic blood pressure monitoring
Falls	Claimed that tamsulosin may cause sedation and advised a decrease in dose when it is, in fact, a single-dose agent. Failed to mention the importance of a multidisciplinary home safety assessment
Dementia	Failed to mention screening for potential substance misuse, e.g., alcohol, which may cause cognitive impairment. Recommended prescribing acetylcholinesterase inhibitors (e.g., Donepezil), however, did not mention the need to exclude a history of bradyarrhythmias
Delirium	Failed to mention the 4AT test or possible delirium triggers like urinary retention or alcohol withdrawal
Frailty	Only mentioned the Fried criteria as an assessment tool for frailty. Failed to mention other tools such as the Clinical Frailty Scale (CFS), electronic frailty index, and PRISMA-7. No mention of the “Timed Up and Go” test. Incomplete nutritional workup. No fall risk assessment was mentioned
Advanced care planning	Overall, high-quality feedback, but failed to mention a lasting power of attorney
Urinary Incontinence	Functional incontinence was omitted, and overflow incontinence was barely mentioned. Overemphasized use of pseudoephedrine for stress incontinence, while failing to mention the use of duloxetine. No mention of screening tools such as the International Consultation on Incontinence Modular Questionnaire

## Discussion

ChatGPT demonstrated generally promising results in simulated geriatric clinical decision-making, with particular strengths in communication clarity and patient safety. The findings suggest that LLMs such as ChatGPT can be a helpful adjunct in supporting clinical reasoning, especially in areas such as education, initial assessment, or when cross-referencing guideline-based care. Reviewers consistently found the responses to be structured, understandable, and often aligned with best practice approaches in geriatric medicine. The consistently high clarity scores likely reflect the model’s inherent design to generate well-structured and confident explanations of existing knowledge, rather than an ability to appropriately weigh clinical uncertainty or real-world risk in complex geriatric decision-making.

However, the study also highlighted important limitations. There were several examples where the AI’s outputs were incomplete or missed critical clinical considerations. For instance, in the delirium vignette, the omission of the 4AT screening tool was noted, and the frailty vignette relied too heavily on the Fried criteria, omitting more widely used tools such as the Clinical Frailty Scale. Similarly, in the discussion of urinary incontinence, pharmacologic recommendations were not aligned with current best practice, and key incontinence subtypes were overlooked. These gaps suggest that while ChatGPT can provide helpful suggestions, it is not yet reliable enough to be used independently in clinical care.

Recent studies across various medical specialties have explored the utility of AI models such as ChatGPT and other LLMs in clinical decision-making. In internal medicine, for example, Kung et al. assessed ChatGPT’s performance on the United States Medical Licensing Examination and found that it achieved passing scores, raising questions about its potential to support reasoning in complex diagnostic tasks [1]. In oncology, Rao et al. demonstrated that AI-generated responses to cancer-related questions were frequently rated as more empathetic and equally or more accurate than those provided by physicians [2]. In radiology and dermatology, similar investigations have evaluated LLMs’ ability to interpret case descriptions and recommend diagnostic approaches, with mixed results depending on task complexity and the specificity of clinical guidelines [3-5]. Notably, Sattler et al. evaluated the diagnostic accuracy of ChatGPT-5 models in distinguishing melanoma in clinical vignettes. While ChatGPT performed reasonably well in broad categorization, the study found its accuracy insufficient for independent diagnostic use, emphasizing the necessity of clinician validation and oversight when integrating AI-generated output into care decisions [6].

Recent evaluations of LLMs have shown rapidly improving performance in medical reasoning tasks. A 2024 systematic review and meta-analysis by Liu et al. demonstrated that GPT-5 achieved an average accuracy of approximately 81% on global medical licensing examinations, markedly higher than GPT-3.5 (≈58%) [7].

Collectively, these findings align with the results of our study. While AI models such as ChatGPT often exhibit strengths in clarity, consistency, and adherence to basic guideline-driven reasoning, they remain vulnerable to omissions, superficial domain understanding, and occasional factual inaccuracies. This underscores the importance of specialty-specific validation, continuous model refinement, and robust clinician oversight before the adoption of AI systems in clinical workflows.

For instance, syndromes such as polypharmacy and falls are well represented in the medical literature, whereas urinary incontinence and advanced care planning involve more contextual or ethical dimensions that may be less consistently documented. These observations support the need for continued validation of LLMs in underrepresented, high complexity clinical domains before integration into routine care [7].

Comparative evaluations of multiple LLMs in clinical recommendation tasks are beginning to emerge. Recent work suggests that performance on clinical recommendation tasks varies across multiple LLMs. For example, Rossettini et al. reported differing levels of agreement with clinical practice guidelines when several LLMs were assessed on recommendations for lumbosacral radicular pain [8]. This highlights the importance of future inter-model benchmarking when interpreting the performance of any single model.

When considering the broader use of OpenAI models in healthcare, their potential is evident, but they must be approached with caution. The free version of ChatGPT, which was used in this study and remains the most commonly accessed by both clinicians and the public, offers widespread availability and can function as a valuable reference point. It performs especially well in summarizing content, offering general advice, and providing structured overviews that can support decision-making. That said, its tendency to occasionally omit key details or present information with excessive confidence, without referencing sources, poses potential risks in a clinical environment. Furthermore, in some cases, the model may generate information that appears plausible but is factually incorrect or misleading, a phenomenon known as “AI hallucination.”

Ultimately, while ChatGPT shows promise as a computer-aided clinical decision support tool in geriatric medicine, particularly for clinicians looking to supplement their knowledge or check against guideline-based practices, it must not replace clinical judgment or structured guideline tools. Its use should always be coupled with critical evaluation by healthcare professionals, and future improvements will be necessary before it can be safely integrated into direct patient care.

Limitations

As only one AI model was tested at a single time point, results may not reflect differences between model versions, prompting strategies, or future updates. Current AI models have a non-deterministic nature, meaning that repeated prompts could yield slightly different responses. Future work should explore response stability and consistency over multiple runs and model updates. Each vignette prompt was submitted once per vignette to reflect typical real-world use. Multiple runs were not performed to assess potential variability in model responses, which may have limited the evaluation of response consistency.

The study included a small sample of vignettes and reviewers. Although inter-rater reliability was high, a broader and more diverse sample would strengthen the generalizability of the findings. The vignettes focused on a single geriatric syndrome; however, it is important to recognize that there are varying degrees of severity within any one syndrome, and clinical presentations do not follow a one-size-fits-all pattern.

Simulated cases may not reflect real-world variability. The use of single syndrome clinical vignettes, while allowing for a standardized and reproducible comparison across domains, may not fully capture the complex, multimorbid presentations typical of geriatric practice. Real-world patients often present with overlapping syndromes (e.g., delirium superimposed on dementia or frailty compounded by polypharmacy), and such interplay can significantly affect reasoning and management priorities. Consequently, ChatGPT’s performance in these vignettes is not necessarily reflective of the day-to-day complexity faced by geriatricians.

Reviewer scoring was inherently subjective, although moderated through independent assessments, use of a predefined scoring framework, and consensus discussion. Similar expert rating approaches have been used in previous studies evaluating digital decision support tools in clinical settings, supporting the validity of this method [1,3]. Nevertheless, some degree of subjectivity cannot be fully excluded. Future work could further reduce this through blinded scoring, larger reviewer panels, and greater use of structured checklist-based assessment.

## Conclusions

ChatGPT demonstrates potential as a supportive tool in geriatric medicine, offering clear and largely safe responses to clinical scenarios. However, variable accuracy and occasional omissions limit its current use in frontline decision-making. Ongoing refinement, integration with validated clinical tools, and closer alignment with evidence-based practices are needed before widespread implementation.
